# Follow-up of health-related physical fitness elements in mild intellectual disability for three years: a sex comparison

**DOI:** 10.7717/peerj.20919

**Published:** 2026-03-04

**Authors:** Murat Ergin, Çalık Veli Koçak, Berkan Bozdağ, Hüseyin Gazi Sönmez, Mustafa Karahan, Umut Canli, Peter Bartik, Peter Sagat, Jason Perez, Pablo Prieto-González

**Affiliations:** 1 Department of Sports Coaching, Aksaray University, Aksaray, Turkey; 2 Department of Physical Education and Sport, Aksaray University, Aksaray, Turkey; 3 Department of Sports Management, Kırşehir Ahi Evran University, Kırşehir, Turkey; 4 Faculty of Humanities and Social Sciences, Abdullah Gül University, Kayseri, Turkey; 5 Department of Sports Coaching, Dokuz Eylül University, İzmir, Turkey; 6 Department of Department of Physical Education and Sport, Tekirdağ Namık Kemal University, Tekirdağ, Turkey; 7 Sport Sciences and Diagnostics Research Group, College of Humanities and Sciences, Prince Sultan University, Riyadh, Saudi Arabia; 8 Preparatory Year Program, College of Sciences and Humanities, Prince Sultan University, Riyadh, Saudi Arabia

**Keywords:** Mild intellectual disabled, Health, Fitness

## Abstract

Children with mild intellectual disability (MID) have significant limitations in both intellectual functioning and cognitive, social, and motor skill behaviors. Understanding the development of physical fitness in boys and girls with MID, and identifying sex-related differences can help devise interventional programs to improve physical fitness in these groups. The aim of this study was to compare sex differences in the time-dependent changes in health-related physical fitness components in individuals with MID. A longitudinal design was employed over three years. A total of 111 individuals with MID (46 girls and 65 boys) aged between 10 and 14 years (mean age 11.97 ± 1.39 years) participated in the study. The physical fitness levels of the participants were assessed using the Brockport Physical Fitness Test (BPFT) battery. The tests included body composition (body height, body mass, and body mass index), aerobic endurance (15 m Progressive Aerobic Cardiovascular Endurance Run (PACER) test), and musculoskeletal function (dominant handgrip strength, back-saver sit-and-reach, and trunk lift). The results revealed that, over time, the longitudinal developmental trajectories for body mass, body height, aerobic endurance, and dominant handgrip strength were more favorable for boys. However, the longitudinal development curves for body mass index (BMI), trunk lift, and flexibility were similar for both boys and girls. The findings of this study provide valuable evidence for developing targeted physical activity programs for individuals with MID, and demonstrate the need for programs aimed at increasing aerobic endurance and muscle strength in girls with MID.

## Introduction

Intellectual disability (ID) is defined as a condition characterized by significant limitations in intellectual functioning and adaptive behavior. It emerges during the developmental period before the age of 22, and manifests in cognitive, social, and practical adaptive skills (Schalock, Luckasson & Tassé, 2021).

Children and adolescents with ID have a higher risk of developing chronic diseases associated with physical inactivity (Rimmer, Rowland & Yamaki, 2007; Winnick & Short, 2014). There are several reasons for the poor fitness levels in children with ID, including sedentary lifestyles (Finlayson et al., 2009; Westendorp et al., 2011), lack of opportunities for sports and physical activity (Baynard et al., 2008), and health-related conditions (Finlayson et al., 2009). Fitness levels in childhood are linked to body mass index (BMI), blood pressure, and cardiovascular health in adulthood (Kvaavik et al., 2009; Sacheck & Hall, 2015). Children with adequate physical fitness have lower metabolic risk factors (Jago et al., 2010), better cardiovascular and musculoskeletal health, lower risk of developing obesity during adolescence (Moreira et al., 2011; Ortega et al., 2011b) and cardiovascular diseases in adulthood (Andersen et al., 2008; Sacheck & Hall, 2015), reduced symptoms of depression and anxiety, and improved academic performance (Ortega et al., 2011a; Ortega et al., 2011b). On the other hand, insufficient physical fitness negatively impacts both physical and cognitive development (Hillman et al., 2009; Pontifex et al., 2011). Moreover, poor physical fitness among children with ID may increase health risks during adolescence and adulthood compared to that in their typically developing (TD) peers (Kristensen et al., 2006).

Therefore, it is crucial to identify the fitness levels of children with ID, and design appropriate physical activity programs to enhance physical fitness. Nevertheless, only a limited number of studies have examined the development of physical fitness in children and adolescents with ID. For example, Baynard et al. (2008) reported that the peak aerobic fitness level in individuals with ID increased between the ages of 9 and 21 years before reaching a plateau. Similarly, Lahtinen, Rintala & Malin (2007) found that abdominal muscle strength and endurance of individuals with ID (IQ range: 30–70) increased between the ages of 11 and 16 years, but later declined in adulthood. However, it is challenging to interpret these cross-sectional comparisons, as children of the same chronological age may differ in biological maturation, leading to significant differences in physical fitness (Flanagan et al., 2015). While the physical fitness levels of TD children are frequently tracked from childhood through adolescence (Kristensen et al., 2006), there is insufficient research on the developmental trajectories of physical fitness in children with MID.

Given the scarcity of longitudinal studies in this population, understanding the time-dependent changes in physical fitness among children with MID, and identifying sex-based differences may help devise effective intervention strategies to improve their fitness levels, and also provide valuable information for families, teachers, and coaches. Therefore, the aim of this study was to examine sex-related differences in the time-dependent changes in physical fitness components among children and adolescents with MID.

## Materials & Methods

### Study design

A longitudinal study was conducted to evaluate the changes in physical fitness levels over three years. In a longitudinal design, repeated measurements of specific variables are taken with the same participants to examine time-dependent changes (Frankel & Wallen, 2012).

### Participants

A total of 144 children who were receiving special education in middle and high schools in Kırıkkale, Türkiye were initially enrolled. The students had been diagnosed with educable ID (IQ score between 50–70) through educational assessment by the Kırıkkale Guidance and Research Center, and placed in mainstreaming or special education classrooms. Official diagnostic reports were obtained annually from the Kırıkkale Guidance and Research Center throughout the three-year study period. The inclusion criteria for the study were as follows: (a) parental informed consent, (b) voluntary participation, (c) no participation in organized sports during the study period, and (d) medical clearance confirming no contraindications to physical activity. The exclusion criteria were as follows: (a) lack of parental informed consent, (b) participation in organized sports during the study period, (c) medical contraindications to physical activity, and (d) the presence of physical disabilities, Down syndrome, autism spectrum disorder, or cardiovascular disease.

The participants only attended 80 min of Physical Education and Sports lecture per week, which was mandatory as part of the school curriculum, and did not partake in any other organized sports activities—either at school or outside. The participants were assessed annually in February for three years (2020, 2021, and 2022). Thirty-three students did not complete the tests due to various reasons, including relocation to another city (*n* = 7), participation in organized sports after the commencement of the study (*n* = 9), inability to participate in the tests due to illness (*n* = 11), and voluntary withdrawal from the study (*n* = 6). The study was finalized with the remaining 111 participants, including 46 girls and 65 boys baseline aged 10–14 years (mean age: 11.97 ± 1.39 years). All data from the 111 participants were included in the statistical analyses. Written informed consent was obtained from the parents or legal guardians of all participants prior to their inclusion in the study.

### Research design

All procedures were approved by the Selçuk University Ethics Committee (approval reference numbers: 2019-73). The physical fitness of the participants was assessed using the Brockport Physical Fitness Test (BPFT) battery (Winnick & Short, 2014), a criterion-referenced test designed to assess health-related physical fitness in children and adolescents (aged 10 to 17 years) with disabilities. Four to six items from the three fitness components (body composition, aerobic function, and musculoskeletal function) are considered general fitness indicators (Winnick & Short, 2014). Seven test items representing three fitness components—body composition, aerobic function, and musculoskeletal function—were selected as indicators of general fitness (Winnick & Short, 2014). During each assessment, the body composition (body height, body mass, and BMI) of the participants was first evaluated, and the tests for musculoskeletal function (handgrip strength, flexibility, and trunk lift) and aerobic endurance (15 m Progressive Aerobic Cardiovascular Endurance Run (PACER) shuttle run) were administered in that order. The participants received two-minute rest periods between the musculoskeletal function tests, and a five-minute rest before the 15 m PACER test. All procedures followed the BPFT guidelines (Winnick & Short, 2014), and the entire protocol required 60–90 min. The participants were given verbal instructions and demonstrations for the tests before starting the evaluation. The tests were administered by researchers specializing in physical education and sports for individuals with special needs, in the multipurpose school gymnasiums of the schools where the students are enrolled. The same researchers conducted the measurements over a three-year period. To reduce measurement bias, data was recorded in a form using anonymized coding of participant names, ensuring all evaluators were unaware of previous results.

### Physical fitness tests

#### Body composition measurements

The height of the participants was measured using a Seca stadiometer (±0.1 cm accuracy), and body mass measurements were taken with the Tanita BC-418MA device (Tokyo, Japan; ±0.1 kg accuracy). BMI was calculated as body mass (kg) divided by height squared (m^2^). For all measurements, the participants were barefoot, wore shorts and a t-shirt, and stood in the anatomical position.

#### Aerobic endurance test

The PACER shuttle run test (15 m or 20 m) is widely used to measure aerobic capacity in children with ID (Collins & Staples, 2017). The test was administered following standardized protocols (Winnick & Short, 2014). To enhance motivation, the researcher ran alongside each participant; however, the pace was set by the participants.

#### Musculoskeletal function tests

Musculoskeletal function includes muscular strength, flexibility, and endurance. In this study, the dominant handgrip strength, back-saver sit-and-reach, and trunk lift tests were conducted following standardized protocols (Winnick & Short, 2014).

#### Dominant grip strength test

This test measures hand and arm strength. Participants sat on a backless chair with feet flat on the floor. The dynamometer was adjusted so that the second phalanx of the fingers rested on the handle. The participants were instructed to squeeze the dynamometer with maximum effort using their dominant hand while keeping the arm away from the body and chair. Two trials were conducted, and the best score was recorded.

#### Back-saver sit-and-reach test

This test is designed to measure hamstring flexibility. The participants removed their shoes and sat on the testing apparatus. They were instructed to extend one leg against the box wall while keeping the other leg bent with the foot flat on the floor beside the knee, and reach forward with both hands (palms down) as far as possible and hold the position for three seconds. The distance was recorded in centimeters. Two trials were performed, and the best score was recorded.

#### Trunk lift test

The trunk lift test is designed to assess trunk strength and flexibility. The participants lay prone on a mat with toes touching the floor and hands under thighs, and were instructed to slowly lift their upper body off the floor while keeping their head aligned and eyes fixed on a predetermined point. The distance from the chin to the floor was measured. For safety, the ruler was placed at least 2.5 cm away from the chin. Two trials were performed, and the best score was recorded.

### Data analysis

Data were analyzed using SPSS software (IBM, version 30, Chicago, IL, USA). The significance level was set at *p* < 0.05. Homogeneity of variance was tested using Levene’s test, and normality was assessed through skewness and kurtosis values. Homogeneity of covariance matrices was evaluated using Box’s test. The test-retest reliability of the measurements was assessed using the intraclass correlation coefficient (ICC), a two-way mixed effects model, with results reported at a 95% confidence interval. A two-factor mixed-design analysis of variance (ANOVA) was conducted to examine the effects of time (T1, T2, T3) as a within-subjects factor and sex (girls and boys) as a between-subjects factor. *Post hoc* comparisons with Bonferroni correction were performed to determine the levels at which differences occurred when significant interaction effects were observed. The assumption of sphericity was tested using Mauchly’s test. When the sphericity assumption was violated, the Greenhouse–Geisser correction was applied. Effect sizes were calculated using partial eta squared (*η*^2^*p*), which indicates the proportion of variance in the dependent variable explained by the independent variable (Fritz, Morris & Richler, 2012). According to Cohen (1988), *η*^2^*p* values between 0.01 and 0.06 indicate a small effect, 0.06−0.14 indicate a medium effect, and ≥0.14 indicate a large effect.

## Results

The Mauchly’s test indicated that the sphericity assumption was violated for the time factor across all tested values (*p* < 0.05). A Greenhouse-Geisser correction was applied since the Epsilon value fell below 0.75. The test-retest reliability for body mass and height was 99% and 98% respectively. The test-retest reliability for BMI was 97%. As shown in to Table 1, there was a statistically significant interaction between time and sex regarding body mass (*F* = 16,81, *p* < 0.05, *η*^2^*p* = 0.134) and height (*F* = 31,7, *p* < 0.05, *η*^2^*p* = 0.225). This finding suggests that the changes in body mass over time differ between female and male participants. The interaction between time and sex for BMI was not statistically significant, and the effect size was negligible (*F* = 0.92, *η*^2^*p* = 0.001).

**Table 1:** Changes in the body composition of the groups over the three-years.

Variables	Groups	Baseline	II	III	Interaction effect (group × time)		
		**Mean ± SD** ***(95% CI)***	**Mean ± SD** ***(95% CI)***	**Mean ± SD** ***(95% CI)***	**F**	***P* value**	***η*^2^ p**
Body mass (kg)	Female	43.62 ± 9.14 *(40.8*−*46.4)*	47.65 ± 9.65 *(44.6*−*50.6)*	51.30 ± 10.05 *(48.1*−*54.4)*	16.818	0.001^*^	0.134
	Male	44.29 ± 9.80 *(41.9*−*46.6)*	49.27 ± 10.87 *(46.7*−*51.8)*	53.05 ± 11.31 *(51.6*−*56.9)*			
Height (cm)	Female	150.78 ± 7.28 *(148.3*−*153.2)*	154.63 ± 6.62 *(152.1*−*157.1)*	157.59 ± 5.77 *(155*−*160.1)*	31.701	0.001^*^	0.225
	Male	149.89 ± 8.99 *(147.8–151.9)*	155.14 ± 9.63 *(153–157.2)*	160.15 ± 8.87 *(157.6*−*162.3)*			
BMI (kg/m^2^)	Female	18.97 ± 2.55 *(18.2*−*19.7)*	19.74 ± 2.74 *(18.9*−*20.5)*	20.5 ± 3.04 *(19.7*−*21.2)*	0.052	0.917	0.000
	Male	19.47 ± 2.52 *(18.8*−*20.09)*	20.22 ± 2.65 *(15.9*−*20.8)*	20.76 ± 2.71 *(20.2*−*21.6)*			

**Notes.**

* *p* < 0.05.

The analysis of test-retest reliability showed high intraclass correlation coefficients (ICC) for all physical fitness tests. As shown in Table 2, the ICC value was 0.94 for the PACER test, 0.98 for handgrip strength, 0.96 for the sit-and-reach test, and 0.97 for the trunk lift test. The PACER test revealed a significant improvement in aerobic performance over time in both groups. The interaction effect for these improvements was also statistically significant (*F* = 3,69, *p* = .038, *η*^2^*p* = .033). In addition, handgrip strength increased for both groups, and a significant interaction effect was observed for this variable as well (*F* = 6,12, *p* = .004, *η*^2^*p* = .053). In contrast, a downward trend over time was noted in both sex for the sit-and-reach test and trunk lift performance. However, the interaction between time and sex was not statistically significant for these tests (*F* = 1,34, *p* = .252, *η*^2^*p* = .013; *F* = 1,496, *p* = .227, *η*^2^*p* = .014, respectively).

**Table 2:** Changes in the physical fitness levels of the groups over the three years.

Variables	Groups	Baseline	II	III	Interaction effect (group × time)		
		**Mean ± SD** ***(95% CI)***	**Mean ± SD** ***(95% CI)***	**Mean ± SD** ***(95% CI)***	**F**	***p*-value**	*η* ^**2**^ **p**
PACER (# laps)	Female	16.09 ± 4.96 *(14.4*–*17.6)*	17.33 ± 5.18 *(15.4*–*19.2)*	19.02 ± 5.70 *(16.7*–*21.3)*	3.694	0.038^*^	0.033
	Male	21.55 ± 5.86 *(20.2*–*22.9)*	23.35 ± 7.41 *(21.7*–*24.9)*	26.57 ± 9.10 *(24.6*–*28.5)*			
Handgrip (kg)	Female	15.34 ± 5.55 *(13.6*–*17)*	16.60 ± 5.90 *(14.7*–*18.4)*	18.04 ± 6.20 *(16*–*19.9)*	6.124	0.004^*^	0.053
	Male	18.65 ± 5.83 *(17.2*–*20)*	20.28 ± 6.83 *(18.6*–*21.8)*	22.57 ± 6.96 *(20.9*–*24.2)*			
Sit and Reach (cm)	Female	23.54 ± 5.12 *(21.7*–*25.3)*	22.00 ± 5.03 *(20.2*–*23.7)*	21.24 ± 4.84 *(19.5*–*22.9)*	1.338	0.252	0.013
	Male	20.37 ± 6.77 *(18.8*–*21.8)*	19.31 ± 6.55 *(17.8*–*20.7)*	17.72 ± 6.44 *(16.2*–*19.1)*			
Trunk lift (cm)	Female	27.91 ± 5.93 *(25.8*–*30)*	26.48 ± 6.05 *(24.4*–*28.4)*	24.98 ± 5.71 *(23*–*26.8)*	1.496	0.227	0.014
	Male	23.83 ± 7.95 *(22*–*25.5)*	21.52 ± 7.28 *(19.8*–*23.1)*	20.68 ± 7.08 *(19*–*22.2)*			

**Notes.**

* *p* < 0.05.

## Discussion

This study examined sex-related differences in the time-dependent changes in physical fitness among children with MID. The findings revealed that boys demonstrated greater improvements than girls in aerobic endurance and dominant handgrip strength. Furthermore, boys showed greater increase in height and body mass relative to the girls. Conversely, BMI, back-saver sit-and-reach, and trunk lift performance exhibited similar trajectories across both sex over time.

A key finding of this study is that the natural development of aerobic endurance in children with MID differs between sex. The small effect size (*η*^2^*p* = .033) suggests that although this difference was statistically significant, individual variation was relatively high in the MID population. Most studies published so far have reported a sex-based difference in aerobic endurance. Some studies show that men perform better than women in both aerobic endurance and muscle strength (Marta et al., 2012; Ortega et al., 2011a; Sauka et al., 2011). This may be related to higher absolute muscle mass and hemoglobin levels in males, as well as sex-based differences in physical activity participation among individuals with MID.

The results of the handgrip strength further indicated that the natural increase in muscle strength in children with MID also follows a sex-specific pattern. Previous studies have shown that individuals with ID tend to have low muscle strength, although males show a greater increase in strength during the growth period (Cowley et al., 2010; Horvat et al., 2019). These differences may be related to hormonal factors, as well as the fact that males exhibit more spontaneous physical activity in daily life and use their upper extremities more frequently. This development is also distinguishable, but to a limited extent, in the MID population, as indicated by the small-to-moderate effect size (*η*^2^*p* = .053).

The superior performance of boys in aerobic endurance and muscular strength may be attributable to higher levels of physical activity participation (Magnusson et al., 2011; Van Sluijs et al., 2021). Studies show that girls have lower levels of physical fitness compared to boys, particularly due to their total body mass (Loftin et al., 2016). In contrast to these findings, Hartman et al. (2015) reported similar developmental trajectories for aerobic endurance and muscular strength in intellectually disabled boys and girls aged 8–12 years. They concluded that the developmental trajectories of physical fitness in children with ID were not affected by sex. This difference may be due to the fact that only children with MID were included in the current study, while Hartman et al. (2015) included children with borderline and moderate intellectual disabilities. Children with MID have lower physical fitness scores compared to children with borderline ID (Golubović et al., 2012; Hartman et al., 2010; Vuijk et al., 2010). In addition, Baranowski et al. (2013) also determined that aerobic endurance develops similarly over time in both boys and girls in sixth and eighth grades.

Muscle strength and endurance are essential for performing daily living activities and participating in leisure activities. Therefore, studies conducted on adults have reported positive correlations between muscle strength, independent living skills, and quality of life (Pillsbury, Oria & Pate, 2013). Current research indicates that girls with MID perform worse than boys in the components of muscular strength and aerobic endurance. This warrants development of fitness and adapted physical education programs to address this deficiency. Furthermore, several studies have compared the physical fitness performance of individuals with disabilities with that of their typically developing peers (Short, 2017). The results indicate that higher levels of physical fitness are associated with greater functional independence, particularly among individuals with disabilities. Biological maturation and pubertal status were not assessed in the present study. Within the same chronological age groups, children who are biologically more mature than their peers tend to demonstrate superior performance in tasks requiring strength, speed, and motor skills (Lloyd et al., 2014). For example, basketball players aged 11–14 years exhibit better physical performance compared to their peers (Guimarães et al., 2019). The quality of force production and neuromuscular structures are among the most important limitations determining the performance of children who are still in the developmental stage (Guimarães et al., 2021).

In contrast to muscular strength and aerobic endurance, flexibility performance over time was similar in both groups, indicating that the change in flexibility in children with MID follows a similar course regardless of sex. Studies show that the flexibility in individuals with ID is generally low to moderate, and that time-dependent change remains limited (Hsu et al., 2021). The slight decrease observed in both groups can be explained by the low physical activity level (Finlayson et al., 2009; Westendorp et al., 2011), limited range of motion, and insufficient muscle–tendon adaptations related to the growth process (Borji et al., 2014), which are common in individuals with MID. The low effect size (*η*^2^p = .013) indicates lack of sex-specific difference in the temporal changes. According to current literature, flexibility decreases with advancing age (Castro-Pinero et al., 2013; Vaquero-Cristóbal et al., 2020). However, the relationship between flexibility and biological maturation remains unclear (Castro-Pinero et al., 2013). Since having a sufficient range of motion in joints is necessary to perform daily activities (Pillsbury, Oria & Pate, 2013), exercise programs that improve flexibility in boys and girls with MID would also be beneficial for functional health. The interaction effect on trunk lift performance was also negligible, which suggests similar time-dependent change in trunk extensor performance in both sex. The decrease in trunk lift performance over time can be explained by the sedentary lifestyle and limited daily activities that adequately stimulate trunk muscles in individuals with ID. Furthermore, trunk muscle performance may frequently be associated with postural control problems and decreased muscle activation in individuals with ID. The low effect size (*η*^2^p = .014) indicates that this change reflects a general temporal trend.

Physiological health is related to both aerobic endurance and body composition. Indicators of body composition, such as skinfold thickness, BMI, and waist circumference, have been associated with risk factors for all-cause mortality, cardiovascular disease, type 2 diabetes, and hypertension in both adolescents and adults (Pillsbury, Oria & Pate, 2013). In this study, the female and male participants showed similar changes in BMI values over time. In contrast, the significant interaction between time and sex regarding body mass and height indicated that time-dependent changes in body mass differ significantly between females and males, most likely due to the sex-related variations in hormones, metabolism, and body composition (Wells, 2007). However, some studies have shown that women with ID are more prone to obesity than their male counterparts (Hsieh, Rimmer & Heller, 2014; Melville et al., 2007). A 30-year longitudinal study by Lahtinen, Rintala & Malin (2007) found that women consistently had higher BMI values than men. This difference was not statistically significant during adolescence, while men had significantly lower BMI values compared to women during adulthood (Lahtinen, Rintala & Malin, 2007). The comorbidity of overweight or obesity in intellectually disabled adults (Jinks, Cotton & Rylance, 2011; Marks, Sisarak & Heller, 2010) begins in early childhood and continues into adulthood (Slevin et al., 2014). Consistent with the current results, Kampasová & Válková (2021) reported similar BMI values for both boys and girls with ID. Furthermore, some studies report that there are no differences in physical performance or body composition variables between the sex during adolescence (Handelsman, 2017; Malina et al., 2010). Biological maturation and age significantly influences athletic performance during adolescence, and the maturation process is responsible for the differences in body composition and physical performance between boys and girls (Handelsman, 2017). Girls reach peak height earlier (9–15 years) than boys (12–16 years) (Malina, Bouchard & Bar-Or, 2004), and the hormonal changes, particularly increased testosterone in boys, are the cause of increase in height (Handelsman, Hirschberg & Bermon, 2018).

This three-year follow-up study provides valuable insights into the natural changes in physical fitness among individuals with MID. However, it has several limitations that should be considered when interpreting the findings. For instance, the absence of information regarding the participants’ engagement in physical activities during the study period restricted the understanding of these changes in physical fitness. Additionally, habitual physical activity levels and participation in organized or informal exercise were not systematically monitored during the follow-up period. Given the wide variability in daily movement commonly observed in individuals with MID, differences in physical activity engagement may have contributed to the observed changes, particularly in aerobic capacity and muscular strength. Another limitation is that maturational status and pubertal development were not assessed. This may be especially relevant for interpreting sex-specific changes over time, as biological maturation is known to influence both aerobic fitness and the development of muscle strength. The field-based tests used in this study showed high test-retest reliability. However, the performance indicators may have been affected by factors such as the participants’ adherence to test instructions, familiarity with the test procedures, and their levels of motivation. These challenges are often encountered, especially in populations with ID. Additionally, the current analysis focused on trends at the group level rather than on individual developmental trajectories. Future longitudinal studies that incorporate objective measures of physical activity, indicators of biological maturation, and suitable comparison groups would provide further clarity on the mechanisms that drive changes in motor function among individuals with MID.

## Conclusions

In conclusion, over the three-year follow-up period, boys with MID demonstrated greater improvements in aerobic endurance and dominant handgrip strength compared to girls. Additionally, the height and body mass of boys also showed greater increase over three years. The developmental trajectories of BMI, flexibility, and trunk lift performance followed similar patterns in boys and girls with MID over time. It is crucial to promote sustained improvements in physical fitness in individuals with ID. The findings of this study provide valuable evidence for developing targeted physical activity programs for individuals with MID, especially programs that improve aerobic endurance and muscular strength in girls with MID. Additionally, further evidence-based research is needed to address and reduce sex-related disparities in physical fitness among individuals with MID.

## Additional Information and Declarations

### Data Availability

The following information was supplied regarding data availability: The raw measurements are available in the Supplementary File.

## Supplemental Information

Supplemental Information 1 Raw data
